# Radiology Residents’ Perceptions of Artificial Intelligence: Nationwide Cross-Sectional Survey Study

**DOI:** 10.2196/48249

**Published:** 2023-10-19

**Authors:** Yanhua Chen, Ziye Wu, Peicheng Wang, Linbo Xie, Mengsha Yan, Maoqing Jiang, Zhenghan Yang, Jianjun Zheng, Jingfeng Zhang, Jiming Zhu

**Affiliations:** 1 Vanke School of Public Health Tsinghua University Beijing China; 2 School of Medicine Tsinghua University Beijing China; 3 Department of Radiology Ningbo No. 2 Hospital Ningbo China; 4 Department of Radiology Beijing Friendship Hospital Capital Medical University Beijing China; 5 Institute for Healthy China Tsinghua University Beijing China

**Keywords:** artificial intelligence, technology acceptance, radiology, residency, perceptions, health care services, resident, residents, perception, adoption, readiness, acceptance, cross sectional, survey

## Abstract

**Background:**

Artificial intelligence (AI) is transforming various fields, with health care, especially diagnostic specialties such as radiology, being a key but controversial battleground. However, there is limited research systematically examining the response of “human intelligence” to AI.

**Objective:**

This study aims to comprehend radiologists’ perceptions regarding AI, including their views on its potential to replace them, its usefulness, and their willingness to accept it. We examine the influence of various factors, encompassing demographic characteristics, working status, psychosocial aspects, personal experience, and contextual factors.

**Methods:**

Between December 1, 2020, and April 30, 2021, a cross-sectional survey was completed by 3666 radiology residents in China. We used multivariable logistic regression models to examine factors and associations, reporting odds ratios (ORs) and 95% CIs.

**Results:**

In summary, radiology residents generally hold a positive attitude toward AI, with 29.90% (1096/3666) agreeing that AI may reduce the demand for radiologists, 72.80% (2669/3666) believing AI improves disease diagnosis, and 78.18% (2866/3666) feeling that radiologists should embrace AI. Several associated factors, including age, gender, education, region, eye strain, working hours, time spent on medical images, resilience, burnout, AI experience, and perceptions of residency support and stress, significantly influence AI attitudes. For instance, burnout symptoms were associated with greater concerns about AI replacement (OR 1.89; *P*<.001), less favorable views on AI usefulness (OR 0.77; *P*=.005), and reduced willingness to use AI (OR 0.71; *P*<.001). Moreover, after adjusting for all other factors, perceived AI replacement (OR 0.81; *P*<.001) and AI usefulness (OR 5.97; *P*<.001) were shown to significantly impact the intention to use AI.

**Conclusions:**

This study profiles radiology residents who are accepting of AI. Our comprehensive findings provide insights for a multidimensional approach to help physicians adapt to AI. Targeted policies, such as digital health care initiatives and medical education, can be developed accordingly.

## Introduction

In this digital era, artificial intelligence (AI) technology is gaining increasing importance in many medical specialties [1,2]. In radiology and other diagnostic imaging–focused specialties, AI has become a more significant lever to help drive high-quality, affordable care [3]. AI is designed to improve health care services while reducing the workload of radiologists and increasing efficiency [4]. In terms of diagnosis, AI aims to help interpret clinical images by identifying specific and complex patterns and providing quantitative evaluation [5]. In general, radiologists are encouraged to get involved in AI [5-7], and those who are open and proactive can be considered early AI adopters [8]. In fact, the advancement of AI will influence the needs and expectations of radiologists, while also presenting new opportunities and challenges within the field [9,10].

Radiologists’ perceptions may influence the actual usage and acceptance of AI technology in clinical practice, and therefore, it is important to understand the variations in perspectives [8,11]. Many studies have explored the acceptance of AI and individuals’ attitudes toward it [12-15]. For instance, a survey in 2018 conducted by the European Society of Radiology reported that only 20% of 675 sampled members were currently using AI applications [16]; a follow-up survey of the same group in 2022 found that 40% of 690 members had experience with AI tools in clinical practice [17]. Previous research demonstrated an overall positive attitude toward AI adoption among radiologists, such as the surveys in Saudi Arabia [18], Ghana [19], and Switzerland [20]. Despite concerns that AI might threaten and replace radiologists [21], a survey conducted across 54 European countries revealed that more than 60% of the 1041 radiologists surveyed responded with “no fear” when asked whether they were concerned that AI would replace their jobs [8]. Another study involving 270 French radiologists found that they disagreed with the notion that AI would replace radiologists [22].

Research suggests that identifying the factors influencing users’ attitudes and acceptance of AI is also important. Previous studies have shown that differences in attitudes toward AI can be ascribed to a wide variety of factors, such as demographic characteristics [23-25] (eg, age, gender, and education), working status [26-29] (eg, occupational health, income level, and job type), psychosocial factors [27], and personal experience [30,31]. AI-related experience is also associated with AI acceptance [32,33]. Contextual factors are considered to play an important role, as learning performance during standardized residency training (SRT) is highly dependent on one’s training status [34,35]. In addition, theories on technology acceptance (eg, the Technology Acceptance Model) suggest that attitudes such as perceived usefulness and risk affect the willingness to adopt AI technology [36-38].

In recent years, the Chinese government’s powerful policies aimed at developing medical AI have effectively promoted the adoption of AI in radiology, especially in diagnosis, disease screening, and prognosis prediction [39]. Before the implementation of AI policies, it is important to investigate physicians’ perceptions and understanding of AI and identify individuals with a proactive attitude as well as the barriers and facilitators to AI acceptance. Moreover, understanding the determinants of their AI acceptance intention is necessary for developing a medical education curriculum and optimizing resident training to facilitate AI competence [40]. Existing research has surveyed Chinese health professionals’ attitudes toward AI in dermatology and ophthalmology, revealing a high level of interest in and acceptance of AI [41,42], whereas empirical evidence for radiologists in China remains limited.

Radiologists have to adapt to the increasing use of AI in their field [7]. Investigating radiology residents’ AI acceptance and associated factors is a precondition. This study aims to extend previous research on AI attitudes, estimate potential predictors of various aspects of AI perception and acceptance, and identify some actionable areas that would inform policy makers nationally and internationally. Based on a nationwide survey of radiology residents in China, this study (1) investigates the perception and acceptance of AI among participants, including their perceived AI replacement, perceived AI usefulness, and AI acceptance; (2) estimates the impact of a wide range of factors such as demography, working status, psychosocial aspects, personal experience, and contextual factors; (3) examines the association between AI perception and acceptance.

## Methods

### Study Design and Participants

This study is a retrospective national survey of radiology residents. The survey was conducted by the Chinese Association of Radiologists (CAR) from December 2020 to April 2021 in 215 cities across 31 provinces in China. To ensure the representativeness of the respondents, the CAR approached all 557 radiology residency programs, and 407 (73.1%) programs were included in the survey. All participants receiving the SRT during the survey period were invited to complete the questionnaire voluntarily and anonymously via “Wenjuanxing” [43], a professional online survey platform. A cover letter stated the purpose of the survey clearly, and the participant-informed consent was obtained before answering the survey. A total of 3666 out of 12,208 potentially eligible radiology residents responded effectively, yielding an overall effective response rate of 30.03%.

### Ethics Approval

Ethical approval was obtained from the Institution Review Board of Tsinghua University (approval number 20210140).

### Measurement

The measures included 6 sections, covering demographic characteristics, working status, psychosocial aspects, personal experience, SRT contextual factors, and perspectives of AI. The study outcomes were binary variables indicating whether participants agreed or disagreed with AI usefulness, AI replacement, and AI acceptance. The CAR survey included 3 items on a 7-point Likert scale for AI perception and acceptance, which have been used in prior research [20,44,45]. Specifically, perceived AI usefulness was assessed by “Do you agree AI helps optimize diagnostic results and reduce errors?”; perceived AI replacement was assessed by “Do you agree that AI will reduce the demand for radiologists?”; AI acceptance was assessed by “Do you agree that radiologists should embrace AI and make good use of it?”. Each question was scored based on the following response options: 1=strongly disagree; 2=disagree; 3=more or less disagree; 4=neutral; 5=more or less agree; 6=agree; and 7=strongly agree.

The demographic characteristics included age (≤27 or >27 years), gender (male or female), educational level (bachelor’s degree, master’s degree, or doctoral degree), and region (east, central, west, and northeast). Working status included eye strain symptoms (frequency of digital eye strain ranges from “never” to “always”), annual after-tax income (≤10,000, 10,001-40,000, 40,001-60,000, or >60,000, in RMB), weekly working hours (≤40, 40-48, or >48), and hours spent on image interpretation per day (<6, 7-9, or >9). Psychosocial aspects considered burnout symptoms (as assessed by the Maslach Burnout Inventory, “yes” or “no”) and psychosocial resilience (as assessed by the Connor Davidson Resilience Scale, ranging from 2 to 14). Personal experience included the experience of working to combat COVID-19 (“yes” or “no”), the experience of making any medical error during the past year (“yes” or “no”), the experience of hearing about AI (“yes” or “no”), and the experience of using AI at work (“yes” or “no”). SRT contextual factors covered SRT training years (the first year, the second year, or the third year), perceptive supports from SRT (as assessed by a 7-point Likert scale), perceptive stress from SRT (as assessed by a 7-point Likert scale), and SRT hospital (“general tertiary hospitals” or “others”). These variables were derived from the CAR survey questions and responses. A detailed description of these measures is provided in Multimedia Appendix 1 [46-51].

### Statistical Analysis

Descriptive statistical analysis was used to calculate the percentage of characteristics among all participants. Means with SDs were presented for continuous variables. The distribution of responses regarding AI-related experience as well as AI perception and acceptance was computed, and mean scores with SD and the proportions of agreed or disagreed were reported. Participants were categorized into 2 groups using mean values as the cutoff. Multivariable logistic regression models were then conducted to identify associated factors of AI replacement, AI usefulness, and AI acceptance. Odds ratios (ORs) and 95% CI were reported. We performed all statistical analyses in Stata (version 17.1; StataCorp LLC). Two-tailed *P* values <.05 were considered statistically significant.

## Results

### Characteristics of Study Participants

A total of 3666 radiology residents were included in this study (Table 1). There were 1539 (41.98%) male residents and 2127 (58.02%) female residents. The mean age of the sample was 27.31 years. Among the residents, 35.05% (n=1285) were in the first year of the SRT program, 96.84% (n=3550) received training in general tertiary hospitals, 40.53% (n=1486) were trained in the Eastern region, 92.06% (n=3375) had a bachelor’s degree, 34.18% (n=1253) reported having earned 10,000 RMBs (after tax; approximately US $1449.8 [US $1=6.8974 RMBs]) or less per year, 56.33% (n=2065) worked 40 hours or less per week, and 79.46% (n=2913) reported spending more than 7 hours per day on image interpretation.

**Table 1:** Baseline characteristics of participants (N=3666).

Variables		Values
**Age (years), mean (SD)**		27.31 (2.58)
	≤27	2228 (60.77)
	>27	1438 (39.23)
**Gender, n (%)**		
	Male	1539 (41.98)
	Female	2127 (58.02)
**Education, n (%)**		
	Bachelor’s degree	3375 (92.06)
	Master’s degree	229 (6.25)
	Doctoral degree	62 (1.69)
**Region, n (%)**		
	East	1486 (40.53)
	Central	742 (20.24)
	West	1220 (33.28)
	Northeast	218 (5.95)
**SRT^a^ hospital, n (%)**		
	General tertiary hospitals	3550 (96.84)
	Others	116 (3.16)
**Annual after-tax income (RMB)^b^, n (%)**		
	≤10,000	1253 (34.18)
	10,001-40,000	880 (24.00)
	40,001-60,000	737 (20.10)
	>60,000	796 (21.71)
**Working hours per week, n (%)**		
	≤40	2065 (56.33)
	41-48	801 (21.85)
	>48	800 (21.82)
**Time spent on image interpretation, n (%)**		
	<6 hours/day	753 (20.54)
	7-9 hours/day	2164 (59.03)
	>9 hours/day	749 (20.43)
**SRT training years, n (%)**		
	The first year	1285 (35.05)
	The second year	1179 (32.16)
	The third year	1202 (32.79)

^a^SRT: standardized residency training.

^b^US $1=6.8974 RMBs at the time of the survey.

### Proportions of AI-Related Experience and Perspectives of AI

Table 2 displays the results of a descriptive analysis of radiology residents’ experiences and perspectives on AI. Nearly 95.77% (3511/3666) of respondents reported that they had heard of AI/machine learning/big data analysis, and 71.99% (2639/3666) had used them at work. Regarding residents’ perception of AI replacing them, 29.90% (1096/3666) of respondents believed that AI would reduce the demand for radiologists. In terms of residents’ perception of AI usefulness, 72.80% (2669/3666) agreed that AI helps optimize diagnostic results and reduce errors. Concerning AI acceptance, 78.18% (2866/3666) of them agreed that radiologists should embrace AI and make good use of it.

**Table 2:** Prevalence of AI^a^-related experience and perspectives of AI (N=3666).

Items	Mean (SD)	Yes/agree^b^, n (%)
1. Have you heard of AI/machine learning/big data analysis?	—^c^	3511 (95.77)
2. Have you used AI/machine learning/big data analysis at work?	—	2639 (71.99)
3. Do you agree that AI will reduce the demand for radiologists?	3.75 (1.53)	1096 (29.90)
4. Do you agree AI helps optimize diagnostic results and reduce errors?	5.27 (1.25)	2669 (72.80)
5. Do you agree that radiologists should embrace AI and make good use of it?	5.45 (1.21)	2866 (78.18)

^a^AI: artificial intelligence.

^b^Experiences of AI were assessed by items 1-2 with a dichotomous response (yes/no). Perceived AI replacement, perceived AI usefulness, and AI acceptance were measured by items 3-5, respectively, with a 7-point Likert scale, and response options higher than “neutral” would be classified into “agree.”

^c^Not available (items 1 and 2 were “yes” or “no” questions).

### Factors Associated With AI Perception and Acceptance

**Figure 1:**
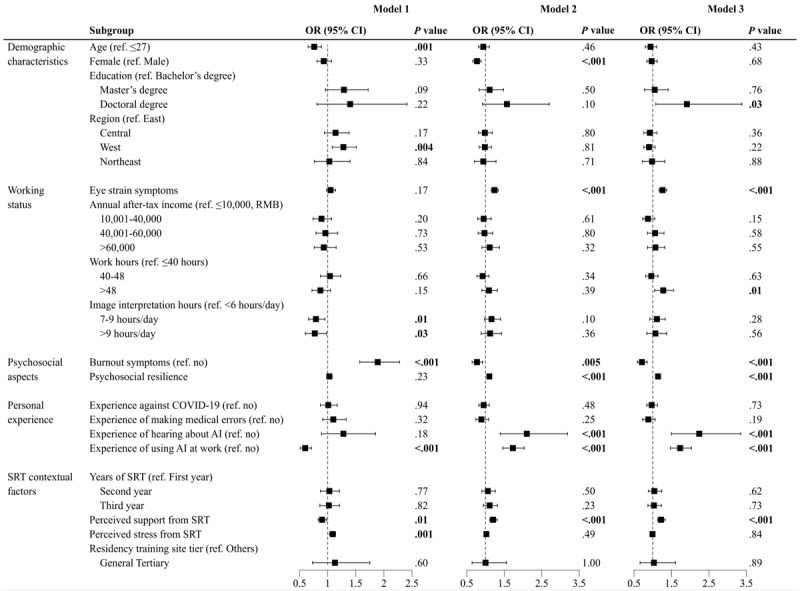
Forest plot of multivariate logistic regression analysis of factors associated with the perceived artificial intelligence (AI) replacement, perceived AI usefulness, and AI acceptance. Associations of multidimensional factors with perceived AI replacement (model 1), perceived AI usefulness (model 2), and AI acceptance (model 3) were analyzed using multivariate logistic regression, respectively. Presented here are the odds ratio (OR; squares) and 95% CI for OR (extending lines). Bold values indicate statistical significance (*P*<.05). For more detailed information, see Multimedia Appendix 2. SRT: standardized residency training.

Figure 1 presents the results of factors associated with AI perception and acceptance from the multivariable logistic regression analysis (also see Multimedia Appendix 2).

In model 1, the results indicated that participants showing potential burnout symptoms (OR 1.89; *P*<.001) and perceiving higher stress levels from the SRT program (OR 1.09; *P*=.001) were more likely to express concerns about AI replacement. By contrast, older participants (OR 0.76; *P*=.001), those spending more time on image interpretation (7-9 hours/day, OR 0.79; *P*=.01 and >9 hours/day, OR 0.77; *P*=.03), individuals who had experience using AI at work (OR 0.60; *P*<.001), and those who perceived more support from the SRT program (OR 0.90; *P*=.01) were less likely to believe that AI would reduce the demand for radiologists.

In model 2, factors associated with the perception of AI usefulness were examined. Respondents experiencing a higher frequency of eye strain (OR 1.24; *P*<.001), possessing greater psychosocial resilience (OR 1.10; *P*<.001), and perceiving more support from the SRT (OR 1.20; *P*<.001) were more positive about the usefulness of AI. Those who had heard about AI (OR 2.10; *P*<.001) and those who used AI at work (OR 1.73; *P*<.001) were more likely to believe that AI could enhance radiology diagnosis. By contrast, female residents (OR 0.77; *P*<.001) and residents with burnout symptoms (OR 0.77; *P*=.005) had less favorable attitudes toward AI’s usefulness.

In model 3, potential predictors of the intention to use AI were higher education levels (doctoral degree, OR 1.91; *P*=.03), a higher frequency of eye strain (OR 1.26; *P*<.001), increased workload (weekly working hours >48 hours, OR 1.28; *P*=.01), higher levels of psychosocial resilience (OR 1.14; *P*<.001), having heard about AI (OR 2.24; *P*<.001), experience in using AI at work (OR 1.73; *P*<.001), and a stronger perception of support from SRT (OR 1.22; *P*<.001). Conversely, burnout symptoms (OR 0.71; *P*<.001) decreased the intention to use AI.

Table 3 demonstrates a significant association between AI perception and the intention to use AI (model 4). In model 5, after adjusting for participants’ demographics, working status, psychosocial aspects, personal experience, and contextual factors, radiologists who perceived higher AI replacement (OR 0.81; *P*<.001) were less inclined to express an intention to use AI, whereas those perceiving higher AI usefulness (OR 5.97; *P*<.001) were more likely to express such an intention.

**Table 3:** Association between AI^a^ perception and intention to use AI (N=3666).

Association					Model 4^b^							Model 5^c^			
					OR^d^ (95% CI)^e^			*P* value			OR (95% CI)				*P* value
**AI perception**															
	Perceived AI replacement			*0.78 (0.73-0.83)*			*<.001*			*0.81 (0.76* *-* *0.87)*				*<.001*	
	Perceived AI usefulness			*6.21 (5.54-6.96)*			*<.001*			*5.97 (5.31* *-* *6.70)*				*<.001*	
**Demographic characteristics**															
	Age (years; reference ≤27)			—^f^			—			1.00 (0.81-1.24)				.97	
	Female (reference: male)			—			—			1.20 (0.99-1.45)				.06	
	**Education (reference: bachelor’s degree)**														
		Master’s degree	—			—			0.88 (0.60-1.28)				.50		
		Doctoral degree	—			—			1.65 (0.82-3.33)				.16		
	**Region (reference: east)**														
		Central	—			—			0.97 (0.76-1.25)				.83		
		West	—			—			0.98 (0.79->1.22)				.85		
		Northeast	—			—			1.00 (0.67-1.51)				.98		
**Working status**															
	Eye strain symptoms			—			—			*1.15 (1.03* *-* *1.27)*				*.01*	
	**Annual after-tax income (reference: ≤10,000, RMB^g^)**														
		10,001-40,000	—			—			0.85 (0.66-1.09)				.20		
		40,001-60,000	—			—			1.05 (0.80-1.38)				.73		
		>60,000	—			—			0.94 (0.71-1.25)				.67		
	**Working hours per week (reference: ≤40 hours)**														
		40-48	—			—			1.01 (0.80-1.27)				.94		
		>48	—			—			*1.47 (1.13* *-* *1.90)*				*.004*		
	**Image interpretation hours (reference: <6 hours/day)**														
		7-9 hours/day	—			—			0.97 (0.76-1.24)				.82		
		>9 hours/day	—			—			0.92 (0.66-1.27)				.60		
**Psychosocial aspects**															
	Burnout symptoms (reference: no)			—			—			*0.78 (0.62* *-* *0.99)*				*.04*	
	Psychosocial resilience			—			—			*1.12 (1.05* *-* *1.19)*				*<.001*	
**Personal experience**															
	Experience against COVID-19 (reference: no)			—			—			0.94 (0.78-1.15)				.56	
	Experience of making medical errors (reference: no)			—			—			0.84 (0.65-1.07)				.16	
	Experience of hearing about AI (reference: no)			—			—			1.28 (0.75-2.19)				.37	
	Experience of using AI at work (reference: no)			—			—			*1.28 (1.03* *-* *1.59)*				*.03*	
**SRT^h^ contextual factors**															
	**Years of SRT (reference: first year)**														
		Second year	—			—			1.04 (0.83-1.30)				.74		
		Third year	—			—			0.91 (0.73-1.14)				.42		
	Perceived support from SRT			—			—			*1.14 (1.02* *-* *1.28)*				*.02*	
	Perceived stress from SRT			—			—			0.99 (0.92-1.07)				.88	
	**Residency training site tier (reference: others)**														
		General tertiary	—			—			0.98 (0.54-1.78)				.94		

^a^AI: artificial intelligence.

^b^Model 4 shows the estimates from the unadjusted model with AI acceptance as the outcome variable.

^c^Model 5 is adjusted for all other factors based on model 4.

^d^OR: odds ratio.

^e^Italics indicates statistical significance (*P*<.05).

^f^Not available.

^g^US $1=6.8974 RMBs at the time of the survey.

^h^SRT: standardized residency training.

## Discussion

### Principal Findings

This study explored the predictors of perception and acceptance of AI technology based on a nationwide sample of radiology residents in China. We found that age, gender, education, region, eye strain status, work hours, time spent on medical images, resilience, burnout, the experience of hearing about AI, the experience of using AI, the perceived SRT support, and the perceived SRT stress have varying effects on diverse attitudes and AI acceptance. Furthermore, residents with positive attitudes toward AI (eg, perceived AI usefulness) have higher intentions to use it, whereas those with negative attitudes (eg, perceived AI replacement) have the opposite effect. Our findings provide empirical evidence for strategies to support the successful implementation of AI in health care settings.

In this study, most respondents had overall positive attitudes toward AI, which is consistent with the results from previous studies [13,52]. Out of the 3666 respondents, 95.77% (n=3511) had heard about AI, and 71.99% (n=2639) had used AI at work. These results align with findings from an international radiologist survey conducted in 2019, where only 4.51% (47/1041) of respondents had never heard about AI [8]. This percentage is notably higher than that of a 2020 survey conducted in Saudi Arabia, where 61.2% (437/714) of radiologists had heard about AI in radiology [53], and it also surpasses the figure from an Australian health care professional survey, where 50% (126/252) of respondents reported current AI usage [54]. The relatively high rate observed in this study may be attributed to the sampled residents receiving SRT in tertiary hospitals equipped with advanced and modern devices. This could be a result of China’s long-term investments in AI-based medical technologies [1]. In our study, 72.80% (2669/3666) of participants deemed AI useful, 78.18% (2866/3666) expressed a willingness to use it, and 29.90% (1096/3666) thought it would reduce the demand for the radiology workforce. This result is consistent with previous studies indicating that the majority of radiologists hold an optimistic view of AI [22] and believe that AI can enhance radiological health care [44], while a minority express concerns about being replaced by AI [8].

Our results confirm earlier findings that older respondents were less likely to agree that AI would reduce the demand for radiologists, while male radiologists were more inclined to believe that AI would benefit diagnosis. Older groups have more work experience and more confidence in their job performance and thus may be less concerned about being replaced by AI [55]. Our study confirms prior observations that males rated AI’s usefulness higher [56]. This suggests that the gender difference observed in this study should be considered when developing an AI-related education curriculum [57]. In parallel, consistent with previous studies [53,58,59], we observed that residents with doctorates were more likely to report an intention to embrace AI in comparison to those with bachelor’s degrees. One possible reason is that higher-educated people have solid theoretical and practical foundations that are ready for AI acceptance. Our findings also reveal a regional disparity in attitudes toward AI replacement. Compared with radiology residents in Eastern China, those from Western China were more likely to be concerned about being replaced, which may be attributed to economic disparity between regions. In China, the western region has consistently lagged in economic development, especially when compared with the more prosperous eastern coastal areas [60-62]. Economic growth boosts labor demand, creating more employment opportunities [63,64], which fosters an individual’s confidence in securing and maintaining a job [65,66]. This finding indicates that the impact of regional economic growth on career confidence should be considered when promoting AI among health professionals.

Importantly, participants experiencing more eye strain tend to view AI positively and support its adoption in the field. This aligns with prior research showing that eye health consciousness positively influences people’s perception of AI’s usefulness. Radiologists experience a higher rate of eye strain symptoms due to their extended periods in front of computers, reading and analyzing medical images [67,68]. AI is expected to expedite scanning, enhance diagnostic accuracy, and reduce radiologists’ workload [69]. Our survey also reveals that radiologists anticipate AI integration to not only improve work efficiency and accuracy but also enhance their health and overall well-being [70].

Radiology residents who frequently worked overtime showed a greater willingness to use AI. In general, the perceived benefits of AI implementation significantly promote its adoption in health care [71], with time-saving through the automation of routine tasks being a prominent advantage of AI applications [72]. Simultaneously, we discovered that individuals who devoted more time to image interpretation expressed fewer concerns about AI replacing them. Medical image interpretation is a fundamental aspect of the radiology profession [73]. Radiologists tend to feel confident and secure when they possess advanced image analysis skills and are recognized as qualified professionals. This finding aligns with a study indicating that IT technicians in hospitals are more likely to hold positive and favorable opinions about AI compared with nurses and doctors [29].

This study empirically supports the association between burnout and AI adoption, demonstrating that burnout is differentially related to various aspects of AI attitudes. Individuals with burnout symptoms were more likely to disagree with the usefulness of AI in health care, show less interest in its adoption, and express concerns about being replaced by AI. People experiencing burnout often report a reduced sense of personal accomplishment [74]. This could explain why those with burnout are more concerned about being replaced by AI. Additionally, burnout has been linked to decreased productivity and career disengagement [75]. Individuals with burnout symptoms may be more likely to exhibit negative attitudes and behaviors in their work, including a reluctance to adopt AI innovations. Conversely, our results demonstrate that individuals with higher levels of resilience hold positive attitudes toward AI, including the belief that AI can improve diagnostic accuracy and a greater willingness to adopt AI. Psychological resilience refers to the ability to adapt to stress effectively [46,76], making individuals with higher resilience better equipped to embrace new technologies. Our findings align with previous research indicating that individuals with high neuroticism, who tend to experience stress in new situations, exhibit more negative emotions toward AI [11]. Prior studies have also suggested that AI adoption holds promise for reducing physician burnout, including among radiologists [77,78]. This study underscores the importance of organizational psychology in promoting AI adoption in health care.

Radiology residents who have heard of AI and used AI at work tend to recognize its usefulness and be more enthusiastic about its adoption. This is consistent with existing research that AI-related background was associated with positive attitudes [79]. People who have used it would agree that radiologists should embrace AI, possibly in large part because it has benefited them. It should be noted that those who had used AI at work were less likely to believe that AI would reduce the demand for radiologists. This may be attributed to the increasing use of AI in the workplace to enhance workflow, allowing radiologists to concentrate more on patient care [80]. For instance, radiology experts believe that AI will not replace radiologists; instead, radiologists who use AI will replace those who do not [81]. By contrast, we found no significant associations between COVID-19 experience or medical error experience and AI acceptance. These results suggest that attitudes toward AI are more influenced by specific personal AI experiences rather than unrelated experiences such as COVID-19 or medical errors. Consequently, targeted AI training programs prove effective in promoting AI adoption among radiologists.

In line with previous research [82,83], our study confirms the significant influence of contextual factors within SRT on radiology residents’ attitudes toward and usage of AI. The 3-year SRT program in hospital settings is a crucial aspect affecting their well-being [84]. Our findings indicate that residents who perceive strong support from SRT are more inclined to see AI as useful, express a desire to adopt it, and have confidence that radiologists will not be replaced by AI. This is in line with previous research showing that perceived organizational support is associated with employees’ perceived usefulness and intention to use a new technology [85]. These results align with the Sociotechnical System Theory, which emphasizes the importance of organizational factors in AI adoption [86]. Furthermore, radiology residents experiencing higher levels of stress in SRT were more likely to believe that AI would reduce the demand for radiologists. This finding can be explained using the job demands-resources model, which categorizes organizational contexts into job demands and job resources. Job-demanding contexts often lead to strain, deplete employees’ energy levels, and elicit negative responses such as job-related anxiety [87,88]. Thus, our findings furnish empirical evidence that support and resources from SRT are crucial elements for facilitating AI adoption [89].

As previous studies have demonstrated, users’ perception of AI significantly influences their intention to adopt the technology [31,90-92]. Our study affirms that perceived usefulness positively affects radiology residents’ intention to adopt AI, while perceived replacement by AI has the opposite effect. Therefore, it is crucial to develop physician guidelines addressing the opportunities and challenges posed by AI to establish a foundational understanding of AI in health care [93,94], including considerations of the legal and ethical challenges associated with AI adoption [95,96].

### Implications

Targeted actions should be taken to promote AI adoption among radiologists. Based on our findings, we recommend specific policy and practice implications. First, considering AI’s potential to reduce radiologist workload [97], integrating AI tools into daily clinical practice could enhance their well-being, especially for those experiencing digital eye symptoms and working longer weekly hours. Second, recognizing the pivotal role of psychological factors such as burnout, policy makers should focus on minimizing potential obstacles to radiology residents’ AI adoption [86], such as addressing the psychological burdens associated with the time-consuming nature of using new technology. Third, to avoid health care inequalities caused by the use of AI technology in health care, curriculum design should take demographic differences in AI acceptance into account [57,98]. Health care management should be aware of the regional gap in AI perceptions. For example, when implementing initiatives to promote AI in economically underdeveloped areas, special consideration should be given to reducing individuals’ concerns about AI replacing them. Fourth, dispelling AI misconceptions and showcasing successful AI adoption cases can help people develop an objective understanding and a positive attitude toward AI applications [99]. Furthermore, involving medical students with AI-related experience in the early stages can boost AI acceptance. Educators can integrate AI teaching into undergraduate medical education and radiology residency curricula to educate students about AI’s benefits and risks, enhancing their theoretical and practical knowledge [13]. Finally, a supportive environment is crucial, necessitating favorable policies to promote AI acceptance and integration, with a focus on fostering a supportive work climate and learning atmosphere.

### Limitations

Several limitations of our study should be noted. First, despite the large sample size, the response rate was relatively low due to the voluntary nature of data collection, potentially introducing selection bias. Second, the use of self-reported data may lead to recall bias, as respondents might provide preselected answers. Third, we used AI as an umbrella term without specifying different types of AI technology in this study, while AI could be categorized based on development stages (eg, strong AI or weak AI) or specific applications (eg, AI-based diagnosis or AI robots). Fourth, due to limited data resources, we only examined 3 aspects of AI acceptance, and future research should investigate additional perceptions and attitudes toward AI (eg, trust in AI). Furthermore, we used a selection of indicators from the CAR survey to align with our research objectives. It is undeniable that better indicators exist for assessing the variable. For a more comprehensive analysis, future studies should consider expanding the survey dimensions to encompass a broader range of associated factors. For example, important work-related dimensions such as the number of cases reviewed per year and the combination of headache and eye strain symptoms should be considered. Fifth, while we used a nationally representative sample, our study primarily focused on a younger group within the medical system, and the representation of senior physicians was insufficient. Future studies should aim to include senior physicians. Finally, it is important to note that causal conclusions cannot be drawn from cross-sectional observational data.

### Conclusion

As AI continues to integrate into health care and daily clinical practice, it is crucial to explore service users’ motivation and engagement to maximize the benefits of new technologies. Building on previous research on AI acceptance, this study provides a comprehensive and nuanced examination of the associations between various antecedents and different AI attitudes, including perceived replacement, perceived usefulness, and acceptance. Based on our nationwide survey in China, this study enhances our understanding of the current state of AI acceptance, especially among Chinese radiologists, the majority of whom are willing to embrace AI. We categorized all associated factors into 5 domains, namely, demographic characteristics, working status, psychosocial aspects, personal experience, and contextual factors. We established 5 models to reveal these complex associations. Our findings suggest that medical educators, hospital managers, and policy makers should be mindful of the barriers and facilitators in promoting AI in health care and develop appropriate procedures and policies. It is essential to adopt multidimensional approaches that involve cooperation across diverse areas, including medical education, hospital management, human resources, organizational psychology, and technology management, to facilitate AI adoption among physicians.

## Appendix

Multimedia Appendix 1 Detailed descriptions on measures.

Multimedia Appendix 2 Factors associated with AI perception and acceptance. AI: artificial intelligence.

## Abbreviations

AI: artificial intelligence
CAR: Chinese Association of Radiologists
OR: odds ratio
SRT: standardized residency training

## Data Availability

The data sets used or analyzed during this study are available from the corresponding author upon reasonable request.
