# Supplementary material for: Radiology Residents’ Perceptions of Artificial Intelligence: Nationwide Cross-Sectional Survey Study
Source: J Med Internet Res. 2023 Oct 19;25:e48249. doi: 10.2196/48249 (PMC10623237; doi:10.2196/48249)
Supplement: Multimedia Appendix 1 [file jmir_v25i1e48249_app1.pdf]

DETAILED DESCRIPTIONS ON MEASURES

Region
The eastern region includes 10 highly developed coastal provincial administrations, including Beijing, Tianjin, Hebei, Shanghai, Jiangsu, Zhejiang, Fujian, Shandong, Guangdong and Hainan. The central region includes 6 provinces, namely Shanxi, Anhui, Jiangxi, Henan, Hubei and Hunan. The western region includes 12 provinces (municipalities), including Inner Mongolia, Guangxi, Chongqing, Sichuan, Guizhou, Yunnan, Tibet, Shaanxi, Gansu, Qinghai, Ningxia and Xinjiang. The northeastern region consists of Liaoning, Jilin and Heilongjiang Province [47].

Eye strain symptoms
The eye strain symptoms indicate the frequency of digital eye strain, which was measured by a single item *How often do you experience eye strain symptoms?* The answer includes 1='never', 2='seldom', 3='sometimes', 4='often', and 5='always', for which the higher scores indicate higher levels of eye strain symptoms.

Burnout symptoms
Burnout was measured with the Chinese version of the Maslach Burnout Inventory-Human Service Survey (MBI-HSS), which has been used in many studies including in Chinese samples [48,49]. MBI-HSS has 22 items and measures burnout from three dimensions: emotional exhaustion (EE, 9 items), depersonalization (DP, 5 items), and reduced personal accomplishment (PA, 8 items). Each item was rated using a 7-point Likert scale of the frequency with choices including 1='never', 2='a few times a year, 3='once a month', 4='a few times a month, 5='once a week', 6='a few times a week', and 7='every day'. For the purpose of this study, we selected 14 items, focusing on domains of EE and DP. Participants with a high score on EE (≥27) or DP (≥10) indicate burnout symptoms.

Psychosocial resilience
Psychosocial resilience was assessed by the 2-item Connor Davidson Resilience Scale (CD-RISC2), which has been applied across diverse populations and showed good psychometric properties consistently [46]. It has also been validated in China and showed acceptable psychometric quality [50,51]. The total score of this scale was calculated by summing the score of each item (from 1 = 'not true at all' to 7 = 'it is true nearly all the time'). The total score ranged from 2 to 14 with a higher score indicating a higher level of psychosocial resilience.

Personal experience
We considered four aspects of personal experience that would impact AI attitudes. The experience of working to combat against COVID-19 was assessed by single question in the survey *Did you participate in efforts related to the fight against COVID-19 (e.g. testing, rescue)?*, and the response options were yes and no. We measured the experience of making medical errors by asking *Have you experienced medical errors that caused harm to patients?*. Meanwhile, the survey also asked two question on AI-related experience, including *Have you heard about AI/machine learning/ big data analysis?* And *Have you used AI/machine learning/big data analysis at work?*.

Perceptive support and stress from SRT

The survey asked participants about their feelings on SRT. The perceived support from SRT was scored by the survey question *Do you agree that you can get enough support from the hospital organization during the SRT program?*, and the perceived stress from SRT was scored by the question *Do you agree that the SRT program's content causes you stress?*. Answers for each questions were measured using a 7-point Likert scale, with seven response options: 1 = 'strongly disagree'; 2 = 'disagree'; 3 = 'more or less disagree'; 4 = 'neutral'; 5 = 'more or less agree'; 6 = 'agree', 7 = 'strongly agree'. The higher scores indicate higher levels of perceived SRT support or perceived SRT stress.
