# Supplementary material for: Radiology Residents’ Perceptions of Artificial Intelligence: Nationwide Cross-Sectional Survey Study
Source: J Med Internet Res. 2023 Oct 19;25:e48249. doi: 10.2196/48249 (PMC10623237; doi:10.2196/48249)
Supplement: Multimedia Appendix 2 [file jmir_v25i1e48249_app2.pdf]

Table S1. Factors associated with AI perception and acceptance.

	Model 1			Model 2			Model 3		
	OR	95% CI	*P* value	OR	95% CI	*P* value	OR	95% CI	*P* value
**Demographic characteristics**									
Age (ref. ≤27)	**0.76**	**0.65 - 0.89**	**.001**	0.94	0.81 - 1.10	.46	0.94	0.80 - 1.10	.43
Female (ref. Male)	0.93	0.81 - 1.07	.33	**0.77**	**0.66 - 0.88**	**<.001**	0.97	0.84 - 1.12	.68
Education (ref. Bachelor's degree)									
Master's degree	1.29	0.96 - 1.72	.09	1.11	0.83 - 1.48	.50	1.05	0.78 - 1.41	.76
Doctoral degree	1.40	0.81 - 2.41	.22	1.57	0.92 - 2.70	.10	**1.91**	**1.08 - 3.38**	**.03**
Region (ref. East)									
Central	1.14	0.94 - 1.38	.17	0.98	0.81 - 1.18	.80	0.92	0.76 - 1.11	.36
West	**1.28**	**1.08 - 1.51**	**.004**	0.98	0.83 - 1.16	.81	0.90	0.76 - 1.06	.22
Northeast	1.03	0.76 - 1.40	.84	0.94	0.70 - 1.28	.71	0.98	0.72 - 1.32	.88
**Working status**									
Eye strain symptoms	1.05	0.98 - 1.14	.17	**1.24**	**1.15 - 1.34**	**<.001**	**1.26**	**1.17 - 1.37**	**<.001**
Annual after-tax income (ref. ≤10,000, RMB)									
10,001-40,000	0.89	0.74 - 1.07	.20	0.95	0.79 - 1.15	.61	0.87	0.73 - 1.05	.15
40,001-60,000	0.96	0.79 - 1.18	.73	0.97	0.80 - 1.19	.80	1.06	0.86 - 1.30	.58
>60,000	0.93	0.76 - 1.15	.53	1.11	0.90 - 1.37	.32	1.07	0.86 - 1.32	.55
Work hours (ref. ≤40 hours)									
40-48	1.04	0.87 - 1.23	.66	0.92	0.77 - 1.09	.34	0.96	0.81 - 1.14	.63
>48	0.87	0.72 - 1.05	.15	1.09	0.90 - 1.32	.39	**1.28**	**1.05 - 1.56**	**.01**
Image interpretation hours (ref. <6 hours/day)									
7-9 hours/day	**0.79**	**0.66 - 0.95**	**.01**	1.16	0.97 - 1.40	.10	1.11	0.92 - 1.33	.28
>9 hours/day	**0.77**	**0.60 - 0.98**	**.03**	1.12	0.88 - 1.43	.36	1.07	0.84 - 1.37	.56
**Psychosocial aspects**									
Burnout symptoms (ref. no)	**1.89**	**1.57 - 2.28**	**<.001**	**0.77**	**0.64 - 0.92**	**.005**	**0.71**	**0.59 - 0.85**	**<.001**
Psychosocial resilience	1.03	0.98 - 1.08	.23	**1.10**	**1.05 - 1.15**	**<.001**	**1.14**	**1.08 - 1.19**	**<.001**
**Personal experience**									
Experience against COVID-19 (ref .no)	1.01	0.87 - 1.17	.94	0.95	0.82 - 1.10	.48	0.97	0.84 - 1.13	.73
Experience of making medical errors (ref. no)	1.10	0.91 - 1.33	.32	0.89	0.74 - 1.08	.25	0.88	0.73 - 1.06	.19
Experience of hearing about AI (ref. no)	1.28	0.89 - 1.85	.18	**2.10**	**1.39 - 3.19**	**<.001**	**2.24**	**1.50 - 3.35**	**<.001**
Experience of using AI at work (ref. no)	**0.60**	**0.51 - 0.71**	**<.001**	**1.73**	**1.46 - 2.03**	**<.001**	**1.73**	**1.47 - 2.03**	**<.001**
**SRT contextual factors**									
Years of SRT (ref. First year)									
Second year	1.03	0.87 - 1.21	.77	1.06	0.90 - 1.26	.50	1.04	0.88 - 1.24	.62
Third year	1.02	0.86 - 1.21	.82	1.11	0.94 - 1.32	.23	1.03	0.87 - 1.23	.73
Perceived support from SRT	**0.90**	**0.83 - 0.98**	**.01**	**1.20**	**1.11 - 1.31**	**<.001**	**1.22**	**1.13 - 1.33**	**<.001**
Perceived stress from SRT	**1.09**	**1.03 - 1.14**	**.001**	1.02	0.97 - 1.07	.49	0.99	0.94 - 1.05	.84
Residency training site tier (ref. Others)									
General Tertiary	1.13	0.73 - 1.75	.60	1.00	0.64 - 1.56	1.00	1.03	0.66 - 1.61	.89

Note: OR = Odds Ratio. Ref. = reference. Boldface indicates statistical significance ($P<.05$). The outcome variables are the perceived AI replacement in model 1, the perceived AI usefulness in model 2, and AI acceptance in model 3.
